# Author’s Response

**DOI:** 10.1308/003588412X13373405385179

**Published:** 2012-09

**Authors:** M Pankhania

**Affiliations:** Milton Keynes General Hospital, Milton Keynes,UK

I thank Professor Stringer for his reply to our technical note, highlighting the significant anatomical variation in scrotal sensory innervation and therefore variable effect with this procedure. His point regarding dual innervation by the ilioinguinal and genitofemoral nerves is particularly pertinent and is an issue that we could not cover fully within the limits of the technical section.

His second point also implicitly demonstrates the risks associated with attempting a regional block of the spermatic cord, which could inadvertently lead to intravascular injection of anaesthetic. We are grateful that these anatomical issues have been brought to wider attention.

